# Dynamic Epitope Expression from Static Cytometry Data: Principles and Reproducibility

**DOI:** 10.1371/journal.pone.0030870

**Published:** 2012-02-08

**Authors:** James W. Jacobberger, Jayant Avva, Sree N. Sreenath, Michael C. Weis, Tammy Stefan

**Affiliations:** 1 Case Comprehensive Cancer Center, Case Western Reserve University, Cleveland, Ohio, United States of America; 2 Department of Electrical Engineering and Computer Sciences, Case Western Reserve University, Cleveland, Ohio, United States of America; Roswell Park Cancer Institute, United States of America

## Abstract

**Background:**

An imprecise quantitative sense for the oscillating levels of proteins and their modifications, interactions, and translocations as a function of the cell cycle is fundamentally important for a cartoon/narrative understanding for how the cell cycle works. Mathematical modeling of the same cartoon/narrative models would be greatly enhanced by an open-ended methodology providing precise quantification of many proteins and their modifications, etc. Here we present methodology that fulfills these features.

**Methodology:**

Multiparametric flow cytometry was performed on Molt4 cells to measure cyclins A2 and B1, phospho-S10-histone H3, DNA content, and light scatter (cell size). The resulting 5 dimensional data were analyzed as a series of bivariate plots to isolate the data as segments of an N-dimensional “worm” through the data space. Sequential, unidirectional regions of the data were used to assemble expression profiles for each parameter as a function of cell frequency.

**Results:**

Analysis of synthesized data in which the true values where known validated the approach. Triplicate experiments demonstrated exceptional reproducibility. Comparison of three triplicate experiments stained by two methods (single cyclin or dual cyclin measurements with common DNA and phospho-histone H3 measurements) supported the feasibility of combining an unlimited number of epitopes through this methodology. The sequential degradations of cyclin A2 followed by cyclin B1 followed by de-phosphorylation of histone H3 were precisely mapped. Finally, a two phase expression rate during interphase for each cyclin was robustly identified.

**Conclusions:**

Very precise, correlated expression profiles for important cell cycle regulating and regulated proteins and their modifications can be produced, limited only by the number of available high-quality antibodies. These profiles can be assembled into large information libraries for calibration and validation of mathematical models.

## Introduction

Investigators often determine expression of various biochemical activities (e.g., protein synthesis; specific phosphorylations) as a function of the cell cycle to obtain an initial indication that the activity has a cell cycle function. For measurement of specific proteins and specific modified protein residues, this is usually accomplished by synchronization, timed sampling, and immunoblotting or immunofluorescence/immunohistochemistry and microscopy. Often, the degree of synchrony and the cell cycle phase distributions within each sample are monitored by DNA content flow cytometry. The same information is available on asynchronous samples using multi-variate cytometry for DNA content coupled with immunofluorescence. Expression of the cyclin proteins as a function of DNA content illustrates the general types of bivariate patterns that result from ∼phase specific oscillating expression [1], [2]. At a glance, the experienced investigator can determine whether expression peaks in G1, S, 4C cells, or at phase interfaces.

Specific mitotic markers are expressed rapidly at the onset of M, and DNA content plus a mitotic marker expands phase resolution by cytometry to G1, S, 4C, and M cells. For somatic cells, adding either cyclin A2 or cyclin B1 to DNA and a mitotic marker expands the phase list to a complete cell cycle: G1, S, G2, and M for the 2C → 4C stemline and the same phases for endo-reduplicated or multi-nucleate sub-cycles [3]-[5].

Generally, the expression of additional markers might be expressed as the average immunofluorescence per each of these phases [6]. However, this view is limited, and we have previously explored the ability to extract expression at high resolution from cytometry data on a relative time basis. In Jacobberger et al. [7], we showed aspects of the basic process, which is to use the correlated expression of DNA and another biomarker to obtain the median sub-population levels of the biomarker as a function of DNA content when DNA is “synthesized” in S phase. This is possible because cytometry of an asynchronous population captures some cells at each point in a programmed process – i.e., incremental levels of the marker map the process. This works for DNA and S phase because DNA synthesis is programmed. A key element demonstrated by Jacobberger et al. is that expression can be mapped in cell cycle phases in which rising or falling markers were not co-expressed, if (1) we have evidence that synthesis or degradation is uni-directional and (2) we have a means to measure the distribution of the marker in a state where expression is essentially not changing and thus, is more narrowly distributed relative to the phases in which levels are changing. In that work, we measured the rising expression of cyclin B1 in G1 and G2 cells by multi-Gaussian modeling, using the distribution in mitotic cells to determine the initial means and standard deviations for the model components. To put the center values determined by each Gaussian component onto an expression versus time profile, the values were plotted in a uniform, incremental manner over hypothetical periods that are typical for the length of G1, S, and G2+M (excluding the degradation of cyclin B1 in late mitosis). While this was an effective means to display the expression features underlying the cytometry data, and provided a method to read fold-expression levels as function of the cell cycle, it was not quantitative with respect to time. Recently, in an effort directed at converting the relative fluorescence data to molecules, we used the frequency information from cytometry data as a surrogate for time [8]. In that study, we converted the median fluorescence within contiguous regions that traced the cell cycle expression “backbone” in bivariate plots of cyclin B1 versus DNA content to numbers of molecules and plotted those values versus the cumulative frequency for all regions encompassing G1, S, G2, and M through metaphase. Since the cell frequency is related to the residence time within any defined region on the bivariate plots for the population at the time of fixation, these plots reflect expression versus cell cycle time. In that work, the focus was on converting fluorescence to molecules and the process of deriving expression versus time was used in its simplest and a statistically loose form. Here, we present a multi-variate analysis that supports complete extraction of expression profiles from the beginning to the end of the cell cycle, improve accuracy, and explore precision of this process. Additionally, we validate multiple sampling, independent analysis, and synthesis of integrated expression profiles for multiple markers as opposed to multi-parametric analysis in which all markers under study are measured simultaneously. This has the distinct value of opening this quantitative approach to an unlimited, N-dimensional biomarker analysis. Throughout the integrated results and discussion, we are oriented to use of this measurement and analysis system to support mathematical cell cycle modeling by systems of ordinary differential equations [9].

Note: in this paper, we refer to 4C cells. These are cells with 4 genome complements. They can be stemline G2 + stemline M + 4C G1 cells of endoreduplicated or bi-nucleate progeny or they can be stemline G2 + 4C G1 cells depending on the bivariate view and/or how the data are gated.

## Results and Discussion

**Figure 1:**
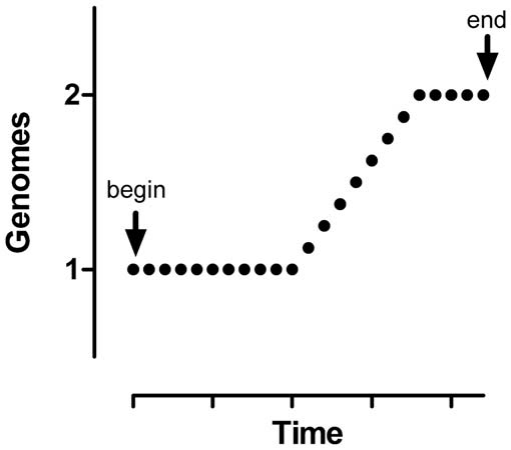
Fundamental Principle of Cytometry. Random sampling from an asynchronous population and measurement (on a cell-by-cell basis) of a parameter that reads out a programmed process populate the programmed expression profile at every point from the beginning of the cell cycle to the end. The example shows a “cell” at each point in time through the programmed expression of DNA content.

**Figure 2:**
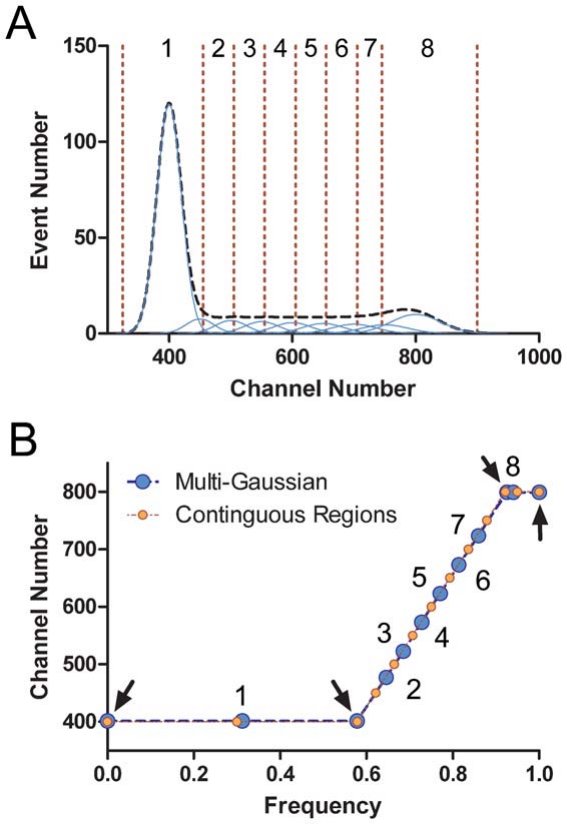
Segmentation of cytometric data. A simulated DNA content distribution composed of the sum (A, dashed black line) of multiple normal Gaussian components (A, thin blue lines) provides a means to extract a perfect DNA content expression profile from idealize typical cytometry histograms (B, blue circles). Segmenting the same histogram by contiguous regions yields the same expression profile (B, orange circles). The segmentation needs to account for measurement variation at the ends of the histogram.

The type of data that encodes complete cell cycle expression information is typified by single cell DNA content measurements from exponentially expanding cell populations. The frequency distribution for DNA content of many individual, asynchronous G1, S, and G2+M cells (perfectly measured) describes the programmed expression for DNA over one expression period, which is the cell cycle time (Tc). Within this program, there are three states, two defined by constant DNA levels that differ by a factor of 2, and one defined by net DNA synthesis. These states are equivalent to kinetically defined G1, S, and G2+M phases. The frequencies of measured G1, S, and G2+M cells are directly proportional to the residence time in each phase or state after adjustment for the age distribution resulting from binary division [10], [11]. **Figure 1** shows a hypothetical expression profile for DNA, typical for cultured mammalian somatic cells, with each simulated cell measurement occupying a discrete position along the program continuum. The length of each of the three segments relative to the abscissa in **Figure 1** is proportional, after adjustment for age distribution, to the size (area) of components in a typical DNA content analysis of an asynchronous cell population measured by cytometry [12]. Histograms of these data are often modeled with three major components: two bounding Gaussian functions and a compound center function that can take several forms with a multi-Gaussian summation being the most versatile [13]. The shape, defined by immediate amplitudes of the sub-component representing the middle segment (S phase) is a function of the rate(s) of DNA synthesis during S phase [12]. The general purpose of modeling histograms is to estimate the number of cells in G1, S, and G2+M in an accurate manner. In a multi-Gaussian model, G1 and G2+M phases are single model components, but S phase is sub-divided into a variable number of sub-components, each defined by a Gaussian function. To extract the expression information from a DNA content histogram, the center values of each component or sub-component are plotted at the center of an interval equal to the fraction of cells in the component/sub-component on a scale of 0–1, with 1 = Tc. **Figure 2A** shows a theoretical distribution built from multiple Gaussian functions that closely matches typical cytometry data.

### Proof-of-principle

To extract expression information from cytometry data, there are two facets to the problem. The first is to define expression over “stretches” of “time” where programmed expression increases or decreases for a single cell as a function of time, and for cytometry data, cells representing each point along that expression curve (see Figure 1) have been measured. Within these “stretches” multi-Gaussian fitting (to account for measurement error) or contiguous arbitrary division of the data into bins (regions) should not be statistically different (since both provide center values for expression and an arbitrary length of Tc fraction based on frequency of measurements associated with that center value. The second facet is that for data regions where the underlying state is constant expression values, the error does have to be estimated and either a Gaussian component fitting or region set to enclose data that vary due to measurement error. Sub-dividing these clusters would lead to erroneous expression curves, since the statistical variation would be measured as either decreases or increases of expression. Both of these facets are illustrated in **Figure 1A** by the vertical lines that define regions or the Gaussian traces (blue) that define both a model fit and the entire distribution (sum of the Gaussians). The only significant difference between an apparent “ideal” approach of multi-Gaussian fitting and the practical, empirical approach of using cursor-delimited regions should be at the major transition boundaries - when the expression slope changes. To test this assertion, we synthesized data similar to that of a typical DNA content histogram and compared the known expression information from the synthetic data to expression extracted from the same data by contiguous regions (**Figure 2A**). This exercise also illustrates the fundamental process of expression extraction. The histogram in **Figure 2A** illustrates a one parameter distribution, simulating a DNA histogram with 60% G1, 30% S, and 10% G2+M. These data were synthesized using 8 normal Gaussian distributions and therefore, the perfect solution for extraction from the composite histogram is a multi-Gaussian fit with single Gaussian components for G1 and G2+M and 6 S phase components (using the same means, standard deviations, and areas that were used to generate the composite). The mean values for each Gaussian component (Y axis) were plotted as described above (**Figure 2B**, blue symbols). For the expression profile to be complete, additional data points were created to occupy the ends (Y intercepts at X = 0 and 1) and to place at the boundaries where expression changed (arrows). The Gaussian components (Figure 2A) and data (Figure 2B) are numbered. The generated points (arrows) were generated by extrapolation. We then analyzed the same histogram using contiguous regions (dashed divisions, **Figure 2A**) and plotted the center values using the same logic in **Figure 2B** (brown/orange symbols). Potentially, the part of this manual procedure where most error could occur is choosing the region boundaries between components (regions 1 and 8). However, the agreement between the manual procedure and the perfect solution is quite good. Therefore, we conclude that we are correct, the error in using the less accurate, more empirical approach is sufficient to obtain the cell cycle related programmed expression of biochemical activities.

The idea of translating cell-based DNA content measurements to an expression trace is obvious from theory [10], [11], but seldom if ever done, likely because the expression program can be imagined accurately and therefore, the need to plot DNA expression doesn't exist. The idea is valid for any other parameter, and for other parameters with more complex expression programs, the need does exist, but the exercise has not been done except for isolated partial attempts [7], [8], [14]. Therefore, we set out to derive the expression of DNA content, cyclin A2, cyclin B1, and phosphorylation of histone H3 (PHH3) from multiparametric cytometry data to explore the problem in multi-parameter data space, to determine the error involved using contiguous regions in multiple dimensions, and to illustrate expression of these three cell cycle programs. To do this, we fixed and stained Molt4 cells for DNA, cyclin A2 or cyclin B1, and PHH3 (3 samples each) and stained 3 samples from the same population for all 4 markers.

### Data preprocessing

There is significant pre-processing of the data prior to the extraction procedure. This consists of aggregate removal, compensation, non-specific antibody back ground subtraction, and rejoining cytokinetic cells (excluded in the aggregate removal step) to the data set. These steps are illustrated in **Figure S1**.

### Unambiguous trajectory

**Figure 3:**
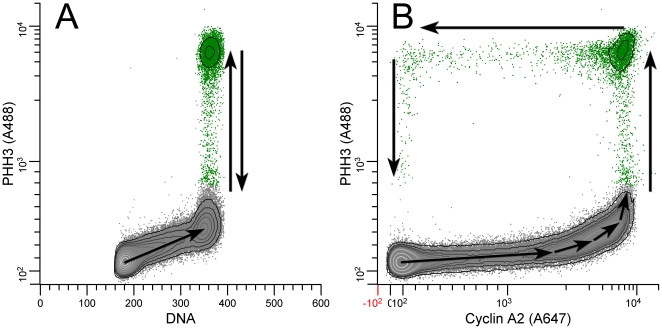
Defining ambiguity. Molt4 cells were stained for phospho-S10-histone H3 (PHH3-A488), cyclin A2 (Cyclin A2-A647), and DNA content. A: typical view for counting mitotic cells (green dots). This view provides ambiguous data vis-à-vis profile extraction since the cells between the G2 cluster and the bulk of mitotic events at the highest PHH3 levels represent cells that were in the processes of net gain and net loss of PHH3 (A, dual arrows). The ambiguity can be resolved by plotting PHH3 versus cyclin A2 (B). Arrows show the direction of movement through the data space of cell traversing the cell cycle.

To perform the same procedure as illustrated for DNA content with more complex data, it is necessary for the analytical setup to provide data that unambiguously captures cells at each point along the expression profile. At a minimum, for each dimension, we need defined variation to create ends for each expression profile, and over any region of each profile, “x”, that oscillates, another parameter, “y”, must either be rising or falling sufficiently to allow separation of the rise and fall of parameter, “x”. The concept is illustrated in **Figure 3**. Expression of PHH3 versus DNA content provides a bivariate view that captures expression in G0/G1, S, G2, and M. However, the mitotic cells that represent rising expression of PHH3 map to the same data space as cells that represent falling expression. The first are at the beginning of mitosis and the last are at the end of mitosis (arrows, **Figure 3A**). In this case, during the period when PHH3 is rising then falling, DNA content does not change, and therefore, the rise and fall of PHH3 cannot be resolved. Addition of cyclin A2 provides a complete, unambiguous data trajectory that can be traced from the beginning of the cell cycle to the end (**Figure 3B**). When PHH3 is rising, so is cyclin A2, but when cyclin A2 is falling, PHH3 is ∼constant, then PHH3 falls when cyclin A2 is essentially nil. Note, however, that this is not independent of DNA content. Figure **S1** shows that segmentation of the 2C stemline from the 4C and higher sub-cycles requires the combination of the three parameters, DNA content, PHH3, and cyclin A2. The unidirectional progression of a hypothetical cell through the data space, as depicted in **Figure 3B** can be verified by monitoring the passage of a BrdU-label through segments of the data space [4].

### Primary segmentation

**Figure 4:**
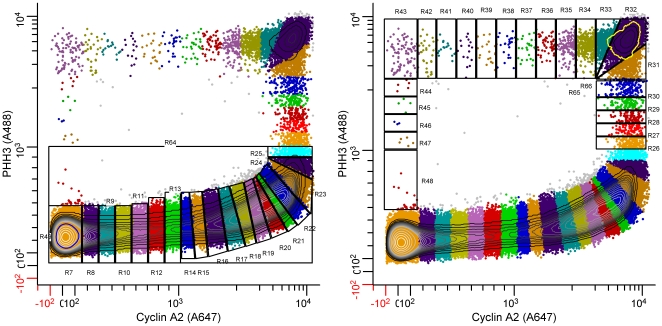
Segmentation in two dimensions. Molt4 cells were stained as in Figure 3. A: segmentation of interphase; B: segmentation of mitosis and the beginning of interphase (R48). For a description of the logic, see the text.

**Figure 5:**
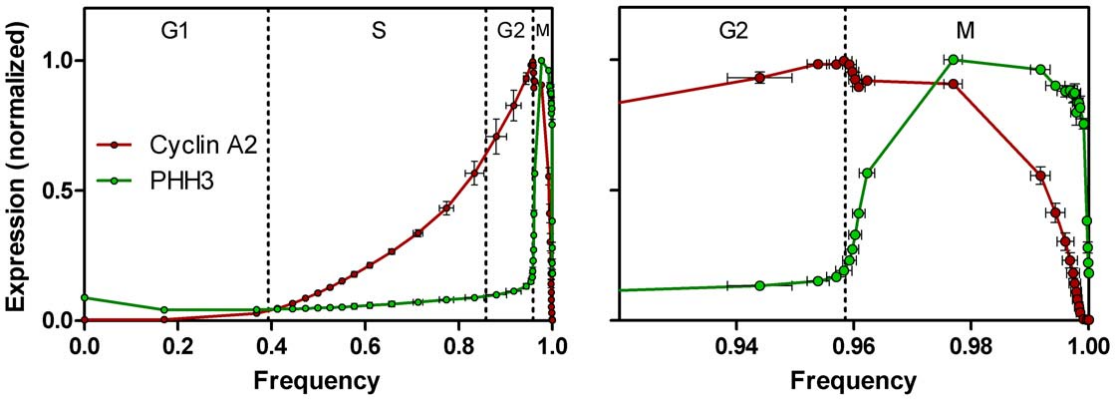
Expression profiles of cyclin A2 and PHH3. Three samples of Molt4 cells from the same culture were fixed, stained (as in Figure 3), and measured independently. Segmentation was as in Figure 4. Expression information and frequency calculations were as described in the text.

**Figure 6:**
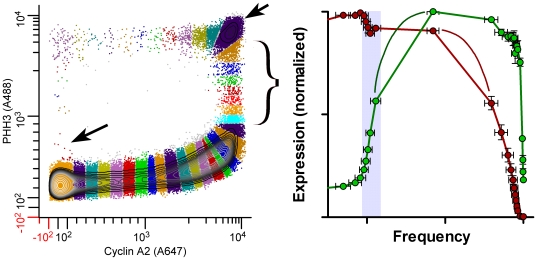
Information details: cyclin A2 and PHH3 expression. Molt 4 cells were stained and analyzed as in Figures 3, 4, and 5. A: brackets show are of cyclin A2 decrease in early mitosis; small arrow points to “new born” cells; large arrow points to large cluster in early mitosis, largely composes of prophase cells. B: gray zone highlights the data derived from bracketed segments in A; small arrow points to data derived from the large mitotic cluster in A; added lines indicate expected expression profile if more data could be obtain within the large cluster in A.

**Figure 7:**
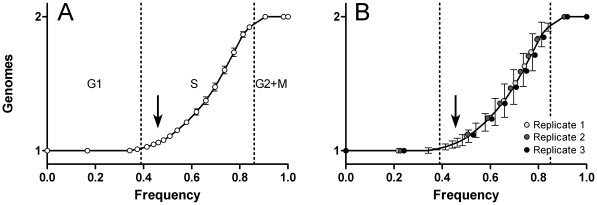
DNA expression profile extraction. A: profile extraction by indirect means. The DNA values were obtained for the cells falling within regions from cyclin A2 vs PHH3 plots as in Figure 4. Error bars are SEM. B: direct extraction compared to indirect. The line traces the data in A; the error bars are the 95% confidence intervals. Circles represent values calculated from each of three Molt4 data sets, derived by directly segmenting DNA histograms as demonstrated in Figure 2.

Expression profile derivation for cyclin A2 and PHH3 can be performed as in **Figure 4**. The approach is to create regions that enclose statistically irreducible clusters (generally, at the ends and corners of the data trajectory) and divide the “stretches” between these clusters into regions with a significant number of events and a goal of providing as many regions within the stretch as is practical. This is valid provided that a cell's residence in any “state” described by a segmented region is dependent on having existed in a previous “state” described by an adjacent region at an immediately earlier time, and that all previous regions are to one side of the immediate region - i.e., the “states” and regions are contiguous, ordered, and unidirectional. This requirement can be satisfied either from direct experimentation - e.g., [4], prior knowledge, or inferred logic. The directionality for cyclin A2 versus PHH3 is shown by arrows in **Figure 3B**. The shape of any bivariate region is conceptually straightforward. The goal is to create adjacent, contiguous “sides” for tri- or tetragons that are perpendicular to a line that follows the local slope of the data. The other two sides are trivial, enclose the data, but could be thought of as parallel to the local slope of the data. This approach is an attempt to assign cells to a level of expression that approximates two-dimensional Gaussian fitting – i.e., account for appropriate variation in two dimensions. The second rule is to bound data at the ends of “stretches” by regions that are not further reducible. For example, R7 in **Figure 4A**, which encloses G1 cells, cannot be made smaller without creating a center statistic for expression that maps variation rather than expression, since that region includes variation that is not further resolvable. This is often not a difficult decision point, since the frequency information, in this case denoted by contour lines, provides information that can be used to determine the best placement of the region boundary. Equally, when the data turn at a right angle, the width of the data in each arm can direct the height and width of the region at the corner. For example, the center and width of the data region bounded by R42 and R44 can guide the size of R43 (**Figure 4B**). In practice, segmenting orthogonal data is very straight forward, whereas data with curvature is problematic because of current software limitations. When data are not orthogonal (e.g., the data captured by regions, R15 through R24, **Figure 4A**), the intent is to follow the two dimensional peak range (i.e., the backbone), distributing the data within a region in a manner perpendicular to the slope. However, we do not know of current software packages that provide the ability to create the desired region shape or eliminate region-to-region overlap with any assurance. For the data presented in Figures 4, 5, 6, 7, to ensure non-overlap and comprehensive inclusion, we calculated the region vertices in a spreadsheet program, then directly modified the “wlx” file (an “XML” descriptor file) in WinList.

### Expression profiles of cyclin A2 and PHH3

**Figure 5** shows the expression profiles of cyclin A2 and PHH3, directly measured from the regions in **Figure 4**. The entire cycle, emphasizing interphase is shown in **Figure 5A**. The detail that occurred in late G2 and mitosis (represented by 4% of the cell population) is shown in **Figure 5B**. These data are the composite results from three independent assays of the same population – i.e., from one batch of fixed cells, three samples were stained and measured and analyzed independently. In this case, the cyclin A2 probe was labeled with Alexa Fluor 647 and the PHH3 probe with Alexa Fluor 488 to minimize spectral overlap. The data set contains staining error and sampling error. The analysis adds analytical error with respect to region size differences. This error is low for measuring center values of expression but larger for establishing the frequency to which a specific level belongs. This is relatively easy to understand. Since the profiles are built on cumulative frequency data, any non-random variation in setting an early region is propagated through the entire curve as a shift. Thus, variation in setting R7 (the G1 region), which is first and contains ∼34% of the events, is propagated as a shift that shows up dramatically at the end of the cell cycle where profile dynamics are abrupt. Nevertheless, the error is acceptable and small relative to the inability to obtain robust profiles by any other currently practiced technology.

### Expression of cyclin A2

The features of cyclin A2 expression are as expected – net synthesis of cyclin A2 beginning at the G1/S boundary. Expression increases in two phases. The first is an approximately linear phase (fits a second order polynomial from Frequency values of 0.38 to 0.7) and the second is exponential (fits an exponential function from Frequency = 0.7 to 0.95). See **Figure S2** for fitting information. There is evidence for two cell cycle transcriptional modes - see references in [15], [16], which may in part explain the two-phase shape of the interphase expression of cyclin A2. In late G2, cyclin A2 expression abruptly peaks and declines as these cells entered mitosis, as determined by elevated PHH3 expression. The abrupt decrease in cyclin A2 levels apparently stabilizes at a new steady state that is maintained for most of the early period of mitosis. We have little detailed information for this period, since the cells are not further divided into individual states and therefore expression is represented by one value. The cells that comprise this period of high cyclin A2 levels are largely prophase cells [17]. In the last quarter of mitosis, cyclin A2 is dramatically degraded to levels that are below our ability to detect by this approach. These interpretations are consistent with current narrative models of cyclin A2 expression in mitosis [18]. This two step decline in cyclin A2 expression, which could be unique to these cells and/or unique to this experiment, is important, since the easiest explanation is that Anaphase Promoting Complex/Cyclosome (APC/C) is activated early but held in check during most of prophase and then dominates after nuclear membrane breakdown. The slight decrease in early prophase may not be an important point for large-view biology, but for mathematical models of cell regulatory biochemistry, subtle results like this, should be useful for model validation. **Figure 6** compares the bivariate histogram and expression data for mitosis. It is significant that even with relatively few cells occupying any one region, the variation in this analysis is relatively low by biology standards. This figure also illustrates that important features like the first period of cyclin A2 decline are readily appreciated from the extracted profile while in the bivariate (in log-log space) are too subtle to be readily noticed. Thus, the act of profile extraction is more instructive than visual inspection of bivariate data.

### Expression of PHH3

Histone H3 is phosphorylated more often at serine 10 when cells are in mitosis. In general, it is a relatively good marker for mitotic cells in that it increases very early in prophase and is still elevated when cells end cytokinesis. This is obvious from the expression profile. Like for cyclin A2, the state wherein PHH3 is maximum and cyclin A2 is at a mitotic plateau (in these cells) is relatively long and uniform in expression of both epitopes, therefore we lose time based expression information. In **Figure 6B**, we have added lines (black) that might be more probable, if we had information at a finer resolution. PHH3 is also expressed above the average G1 level in a small subset of cells in G1 (arrow, **Figure 6A**). These are cells that have recently divided. This population correlates with a similar population marked by an antibody reactive with phospho-S780-Rb (unpublished data), and that state displays BrdU-labeled cells prior to the bulk of G1 cells in kinetic experiments [4].

### DNA content and indirect profile derivation

In principle, it is possible to derive additional parameter expression profiles from the regions set up as in **Figure 4**, since that set of regions unambiguously traverses the entire cell cycle. The caveat is that any expression profile that is derived should not oscillate within any single region, since this would create ambiguity for that parameter. Additionally, there is a problem with using regions set up on a bivariate to indirectly derive third parameter expression. The data trajectory can be visualized as a worm contorted through three dimensional data space. Regions set up on two parameters are unlikely to appropriately have the correct orientation with respect to the three dimensional trajectory, since current software correlates the third parameter orthogonal to the plane of the bivariate within the bivariate region. To ask whether we can accept the error involved, we derived the expression profile for DNA content from the regions set as in **Figure 4** and compared the results to expression profile data for each replicate, derived from single parameter DNA histograms as in **Figure 2**. The results are shown in **Figure 7**. It is clear that in this instance, the error was acceptable. **Figure 7A** shows the DNA expression profile derived indirectly with error bars equal to SEM. **Figure 7B** shows the same plot without symbols and error bars equal to the 95% confidence intervals. The data are from single dimension histograms for each replicate, analyzed independently - i.e., there is no relationship to the regions between analyses other than that nine regions were used in each instance. It is clear that the directly derived profiles (**Figure 7B**) and the profile derived indirectly agree exceptionally well. An added benefit to the indirect method (in this case) is that the boundary at G1/S (arrows) is better defined because immunofluorescent detection of cyclin A2 is a more sensitive marker for S phase than is DNA content measured by dye fluorescence.

### Cyclin B1 expression

**Figure 8:**
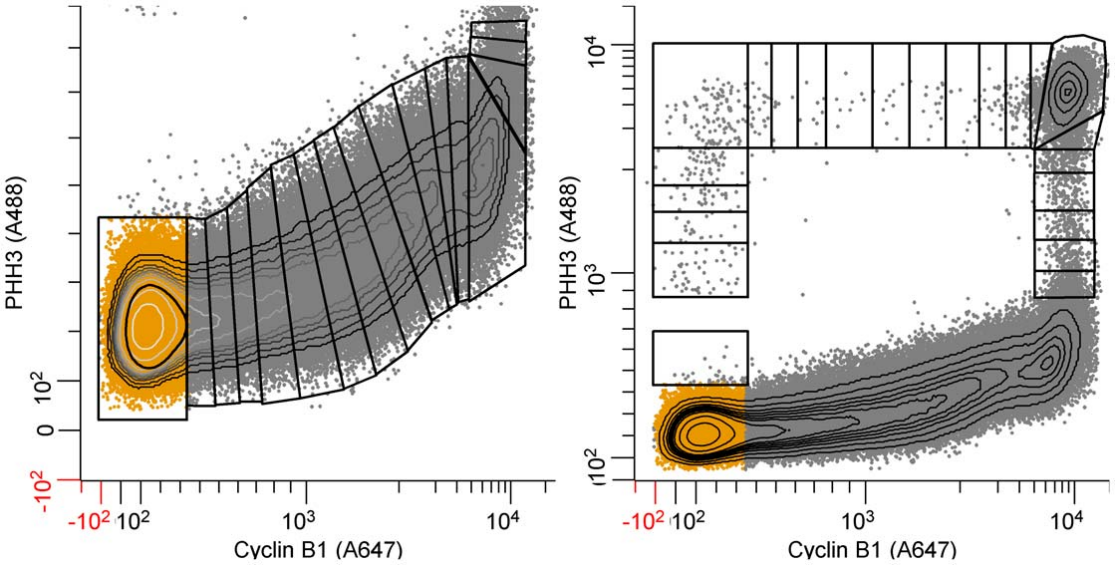
Segmentation for cyclin B1 and PHH3. The approach is as in Figure 4 with differences due to the pattern created by the relationship between cyclin B1 and PHH3. A:interphase segmentation. B: mitotic segmentation.

**Figure 9:**
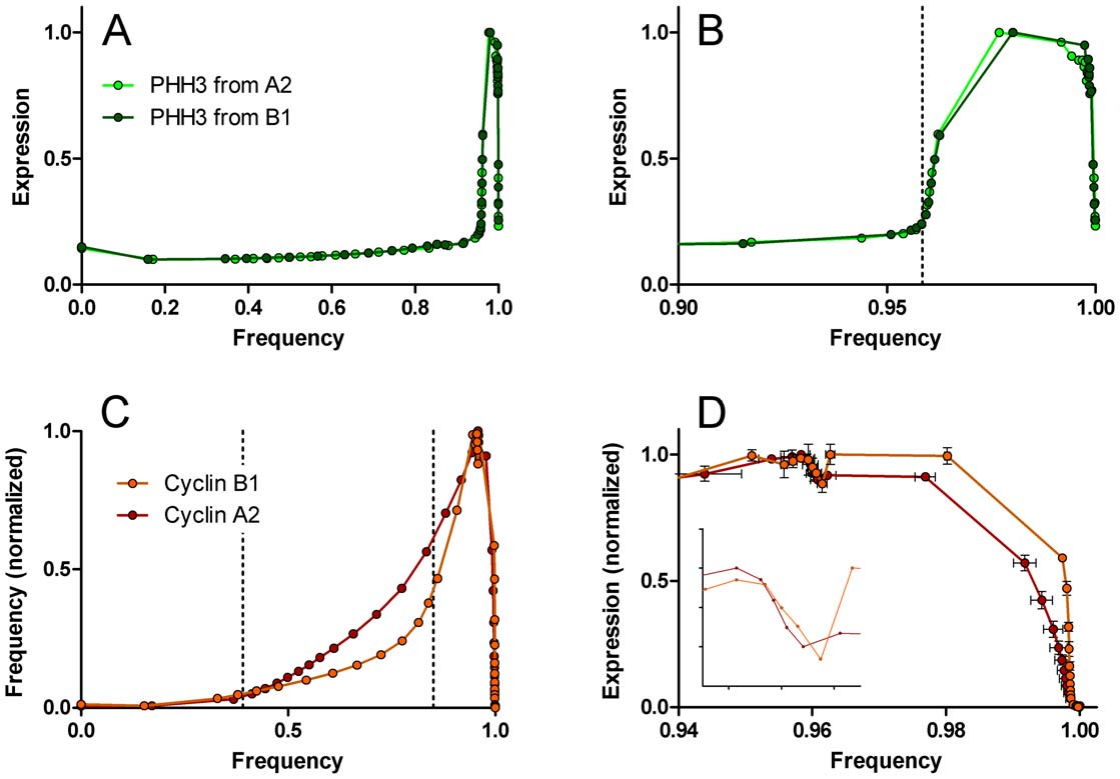
Overlayed expression profiles of cyclins A2 and B1. Overlays of expression profiles for PHH3 (A, B) from the data set Molt4 cells stained for cyclin A2, PHH3, and DNA content (labeled PHH3 from A2) presented in Figure 5, and from cells stained for cyclin B1, PHH3, and DNA content (labeled PHH3 from B1). C, D: overlays of expression profiles for cyclin A2 and cyclin B1 obtained independently on the same samples. Inset in D: zoomed view of initial decrease in both cyclins as cells enter mitosis. In A, B, and C, means (of median specific fluorescence) are shown. In D, error bars are SEM.

**Figure 10:**
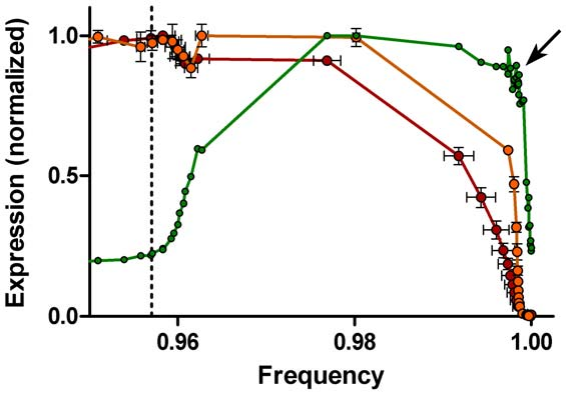
Joined expression profiles for PHH3, and overlayed profiles for cyclins A2 and B1: mitotic view. For PHH3, means values are shown as in Figure 9, but each data set has been joined. The data density along the profile is enhanced; the arrow points to region of obviously enhanced data density.

In this series of stained samples, the same population of Molt4 cells used for cyclin A2 and PHH3 (above) were stained for cyclin B1, PHH3, and DNA content. The purpose was to obtain the cleanest measure of cyclin B1 (i.e., without fluorescence compensation) and to test the ability to measure cyclins A2 and B1 independently and put them back together through the frequency domain. Figure 8 shows segmentation of flow cytometry data for cyclin B1 versus PHH3 for a sample from the same population of Molt4 cells for which the cyclin A2 analyses were made. Essentially, the cytometric data patterns look similar from this view, reflecting the similar shapes of the expression profiles for these two cell cycle regulated proteins. We can measure cyclin B1 in G1, earlier than cyclin A2, and the subsequent rise cyclin B1, peaking at the G2/M transition, can be fit by a compound equation with two exponential components (**Figure S2**). Apparently, both cyclins are expressed at one rate through S and a second, increased rate in G2, with cyclin B1 affected more dramatically. This comparison is shown in **Figure 9C**. In **Figure 9**, the data sets for cyclin A2 and cyclin B1 (3 independently analyzed samples of each) are co-plotted. In **Figure 9A/9B**, the PHH3 profiles from the two data sets are co-plotted, demonstrating remarkable accuracy for two independently stained and measured samples, and independent analyses of the two data sets. The agreement in the two PHH3 profiles validates co-plotting of the cyclins with the features of each cyclin, relative to the other, valid as well. As independent confirmation, the analysis shows that cyclin A2 expression decreases to background levels prior to a similar decrease in cyclin B1 (**Figure 9D**), which is known to be the case [18]. **Figure 9D** (inset) shows that both sets of data indicate a decrease in both cyclins at the same time in the beginning of M when PHH3 is rising. This adds weight the idea that this is a real event, at least for this cell line under the culture conditions at the time of the experiment, and if more generally observed, should eventually be accounted in mathematical models of the biochemistry of mitotic entry. The data presented in Figure 9 show that by the expression profile extraction approach, the relative expression behavior of an essentially unlimited number of biomarkers can be assessed as a function of the cell cycle by independent sampling, measurement, and analysis, and quantitatively put together as a whole analysis. This then becomes a powerful approach to validating mathematical models of the cell cycle. **Figure 10** shows a close-up of mitosis from the view of both analyses. In this figure, the means for the two sets of PHH3 expression data are plotted as one set, demonstrating an increase in information by (1) more points defining PHH3 during the periods of cyclin degradation (arrow) and (2) the temporal offsets of cyclins A2 and B1 degradation and PHH3 dephosphorylation.

### Comparison of unified and independent analyses

**Figure 11:**
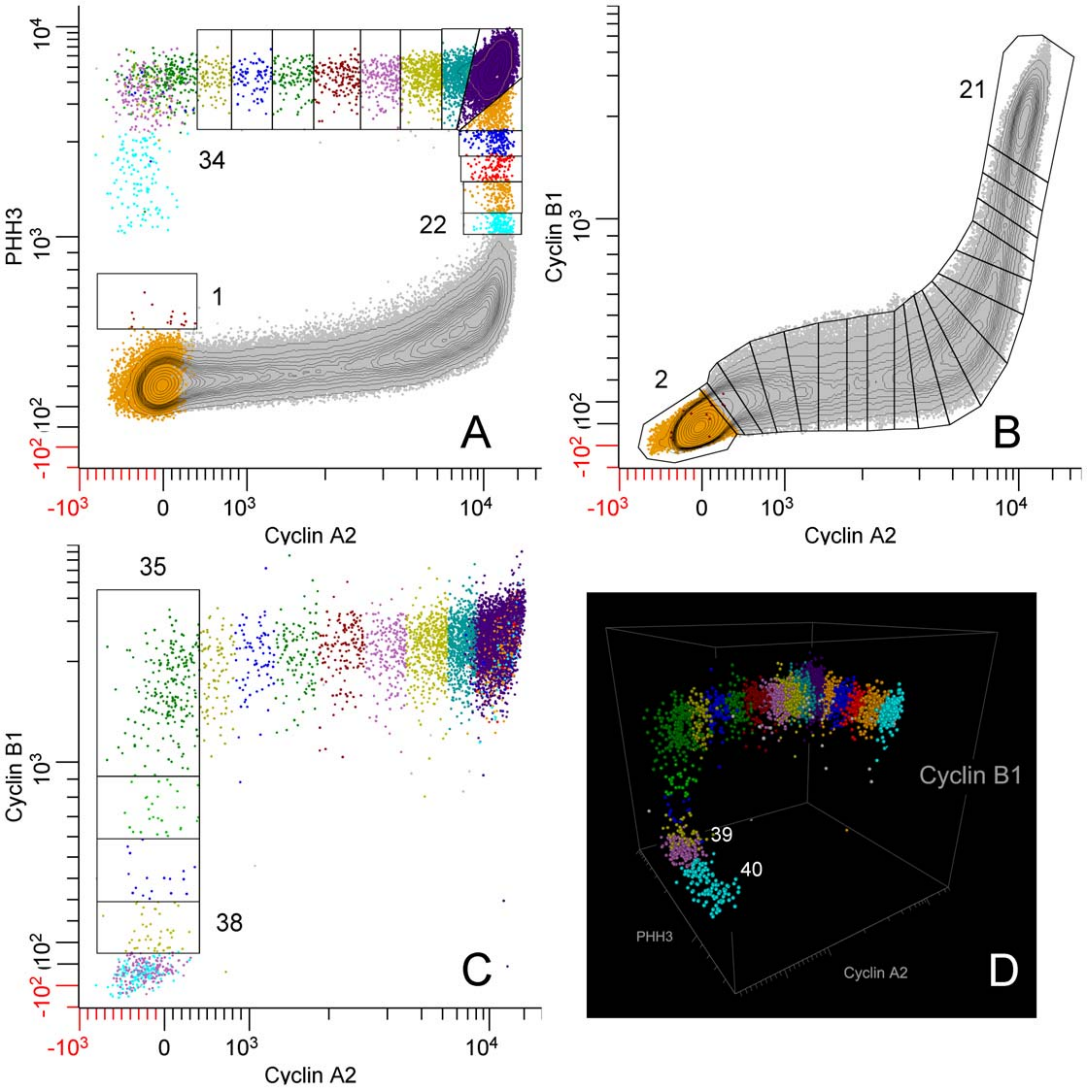
Segmentation of multiparametric data. Samples from the same Molt4 population as shown in previous figures were stained for cyclins A2, B1, PHH3, and DNA content. Segmentation is more complex then single cyclin stained samples, requiring four bivariate views. The first segment (labeled “1”) begins in A (cyclin A2 vs. PHH3) then jumps to B, which is a plot of cyclin A2 vs. cyclin B1 for interphase cells (NOT gated for mitotic cells identified by a region (not shown) set as in Figure S1), and begins with “2” and ends with “21”. The next segment is back in A, labeled “22” and ends at “34”. The next segment, “35” is in C, which is a plot of cyclin A2 vs. cyclin B1 for mitotic cells (AND gated for mitotic cells as in Figure S1). The final cluster is segmented in a bivariate of PHH3 vs. cyclin B1 (not shown). D shows resolution of the last mitotic cluster by color (cyan, magenta), labeled “39” and “40” in a 3D plot of PHH3, cyclin A2, and cyclin B1 to show the orthogonal nature of the segment continuum for mitotic cells.

**Figure 12:**
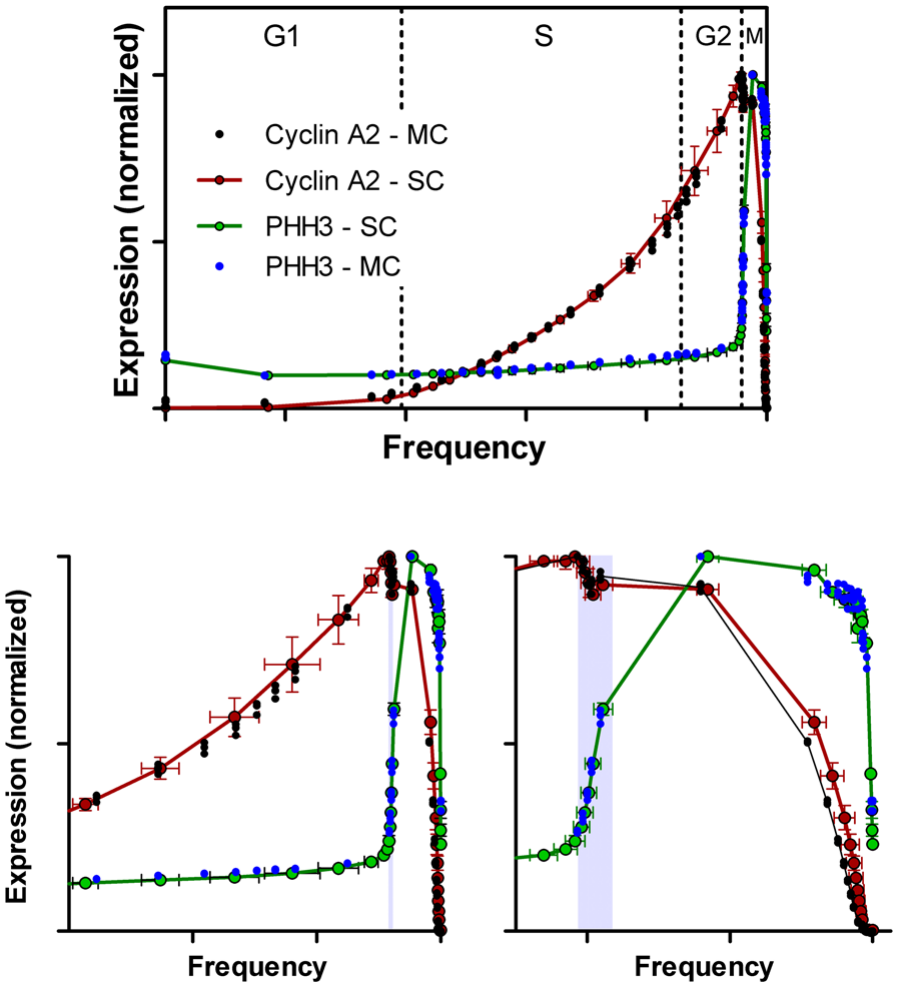
Expression profiles from multi-parametric data. PHH3 (green) and cyclin A2 (red) profiles, extracted from single cyclin sample data, are co-plotted with PHH3 (blue) and cyclin A2, extracted from multi-cyclin sample data, in three views that emphasize the entire cell cycle (top), G2 and M (lower left) and mitosis (lower right).

**Figure 13:**
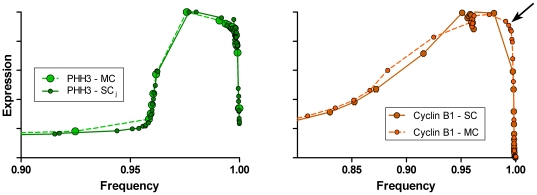
Expression profiles from multiparametric data. Joined PHH3 profiles extracted from single cyclin sample data (both cyclins A2 and B1) and multi-cyclin data are co-plotted (left). A cyclin B1 expression profile extracted from single cyclin data (SC) is co-plotted with an expression profile extracted from multi-cyclin data (MC).

Improvements in instrumentation, antibody specificity and affinity, and fluorescence labeling probes have colluded in recent years to push the possibility of multiparameter cytometric analyses to 17 or more biomarkers [19]. However, fluorescence spectral overlap and the general non-availability of labeled antibodies often reduce the practical number of simultaneous biomarkers to much less than this. In analyses like we have presented here, where expression does not equal well-separated clusters of “negative” and “positive” cells but rather forms a continuum from low to high expression, the spectral overlap problems are even more inhibitory. This is something that we have suspected or known, but is difficult to demonstrate. We performed the measurements and analyses presented in **Figures 11** **,** **12** **,** **13** to (1) further validate that the composite analyses in Figure 10 was correct, and (2) to evaluate the impact of spectral compensation for the overlap of Alexa Fluor 488 (on the PHH3 antibody) and phycoerythrin (on the cyclin A2 antibody). Three samples from the same population of cells used to generate the “single color” cyclins A2 and B1 data were stained for cyclin B1 (Alexa Fluor 647), cyclin A2 (phycoerythrin), PHH3 (Alexa Fluor 488), and DNA content (DAPI). Bivariate plots of the resulting data for one sample, and the segmentation scheme are shown in **Figure 11**. We used a different segmentation scheme that takes advantage of the correlation between cyclins A2 and B1 and the early rise of cyclin B1 to produce a smaller base cluster in G1 (orange dots, **Figure 11B**) and to ask whether a different segmentation scheme affected the data. Additionally, since cyclins A2 and B1 were correlated, we could dissect mitosis in more detail, examining the periods of time when first cyclin A2 and then cyclin B1 is degraded. The segmentation starts in **Figure 11A** with the region labeled “1”, then jumps to **Figure 11B** to region “2”. The consecutive regions move unidirectionally from “2” to “21” following the bivariate synthesis pattern of the two cyclins in interphase. The next region, “22”, appears in Figure 11A with consecutive, unlabeled regions ending at “34”. This region set is very similar to the mitotic regions in **Figure 4B**. The next region, “35”, starts in Figure 11C and ends just before the terminal cluster in this bivariate plot. Regions “39” and “40” finish the sequence and are shown in the three dimension view of PHH3, cyclin B1, and cyclin A2 expression in mitosis (**Figure 11D**). The expression profile data for each of the three measured samples are plotted for cyclin A2 (black circles) and PHH3 (blue circles) with the “single color” data of **Figure 5** (red and green data) in **Figure 12**. It is obvious the PHH3 data are not different and for interphase and early mitosis, the cyclin A2 data match as well. However, the cyclin A2 data appear to be lower in values than expected (from “single color” data) when cyclin A2 was actively degraded in mitosis. This is also the data that are most sensitive to spectral compensation problems and where event numbers are small. Co-plotting of the multi-color (MC) cyclin B1 data with “single color” (SC) data in **Figure 13B** show that cyclin B1 was not subject to the same errors. The cyclin B1 probe, labeled with Alexa Fluor 647 was not subject to spectral overlap problems and compensation was not employed. **Figure 13A** shows the overlay of multi-color PHH3 with single color PHH3 from the cyclin B1 series of samples and shows identity. The payoff from multiparametric data is enhanced clarity of information as shown from the cyclin B1 values (**Figure 13B**, arrow) that were obtained indirectly from the regions set on the cyclin A2 versus PHH3 plot on the region that defines the period of cyclin A2 degradation. These measurements are not possible in “single color” cyclin B1 data.

### Additional Discussion

Here we presented the case for extraction of the cell cycle expression of biomarker sets defined by antibody reactivity and DNA binding dyes that requires two antibodies, reactive with one mitotic cyclin and one marker that is expressed highly in mitosis, and the DNA dye, and at least one other marker of interest. Analytically, this system of measures and analysis has no limits provided that multi-variate data can be obtained that “worms” unidirectionally through multi-variate data space unambiguously (in the simplest version, that is what a mitotic cyclin, a mitotic marker, and DNA content provide). Practically, the system is limited by the availability of high quality (high specificity and high affinity) antibodies. Nevertheless, the current catalog of readily available probes that meet these requirements is quite large, and significant studies could be designed to capture expression data for a large set of cell cycle related epitopes.

Aside from natural curiosity, our current drive for this work is to provide a means to validate mathematical models of the cell cycle based on systems of ordinary differential equations (unpublished data). As presented here, there are two problems that we have not addressed. The first is that this measurement system produces correlated, relative expression data that provides timing information but not data in a final form that reflects expression molecule for molecule. The second is that use of cell frequency as a surrogate for time needs an additional transformation to account for the one mitotic cell giving rise to two G1 cells. Each of these problems can be addressed, and we have addressed them in other publications. To address the first, we used the same example measurement system as this communication, but used the “single color” files to generate a relative relationship between expression of one epitope and expression of a second (in the example, cyclins A2 and B1). That relationship was used to transform multi-variate data to the same relative scale (unpublished data). To transform those relative scales to a molecular scale, quantitative western blotting with purified molecular standards, as we have previously published [8], [20], can be employed. This approach is not exact - it is dependent on a high level of procedural rigor, and the antibodies should have similar affinities and be of the same isotype. Error is introduced by unequal affinities, differential recognition by the secondary antibody, and any differences in epitope masking. Nevertheless, this approach is a good and feasible first step toward production of a model based on reality.

We used a concrete, but unsophisticated method for extracting the expression information from cytometry data. First, we used one or more bivariate plots to segment the data based on the local bivariate slopes along a twisting data path. Aside from the simple approach to chopping Gaussian distributed data into blocks, the main problem with this approach is that any other parameters are necessarily assumed to be orthogonal in the 3rd dimension relative to the X and Y of the bivariate plot. They often are not. However, we showed in this paper that we don't seem to pay a large penalty for this. Most likely, this is because we are measuring centers and over the entire span of the N dimensional data, the imprecision in this approach has a small effect. Whatever the reason, empirically, we have shown that this approach works. The reason that we chose this approach is that current cytometry software is severely limited with respect to analyzing multidimensional continuums. There are two steps toward a more sophisticated approach. The first is the create software with N-dimensional region setting. A significant problem is that analyses of this type of data are almost completely visually guided and exactly how to approach dimensionality past three is not clear. A next, second step would be to write N-dimensional Gaussian component modeling. We have used this approach in single dimensions with good results [8], [21]. The problems with this is that most immunofluorescence distributions approximate normal or log-normal distributions, but often not exactly, and often in parts of the data path, the number of events populating a region may be too low for good fits, especially in N dimensions. Further, it is unclear at the outset that the penalty that any gains in accuracy will offset the relative robustness and reproducibility of the current approach. Currently, writing software for multi-dimensional analysis is an expensive and time-intensive undertaking. The value of the current approach is that with minor modifications (chiefly, easy insurance that regions don't under- or over-lap each other), current software could be revised to make this approach easily accessible. An intriguing new development is embodied by a concept termed “probability state modeling” [22]. In this system, data are clustered along profiles that, for the type of work presented here, look exactly like expression profiles. Multi-dimensional data are analyzed by assigning data to sequential profiles and the correlated nature of the data sequentially restricts the data to regions along the profile X axis until uncertainties are minimized. The result is the best partitioning of the data along an expression profile that is statistically supported in a rigorous and sophisticated manner. It seems to us that this approach is the right direction for the type of analysis presented here, however, it is difficult to see at this time how that could be implemented de novo (without the prior expression profile knowledge).

## Materials and Methods

### Cell Culture

Molt4 cells ([23]; obtained from Keith Shults) were cultured in RMPI 1640 medium without phenol red with 10% fetal bovine serum and gentamycin at 37°C with 5% CO_2_ in T75 flasks. Cells were split approximately twice per week to maintain concentrations between 3×10^5^–1×10^6^. Samples were from exponentially growing cultures.

### Cell Fixation and staining

Samples were washed and fixed with formaldehyde and methanol, stored at −20°C until staining, then stained for cyclin A2, cyclin B1, PHH3, and DNA content as previously published in detail [24]. Three samples were stained simultaneously for cyclin A2, PHH3, and DNA and three samples were stained for cyclin B1, PHH3, and DNA by indirect method for each cyclin. A third set of three samples was stained with directly conjugated antibodies for all three epitopes and DNA content as in indirect samples.

### Antibodies and dyes

2×10^6^ fixed cells per sample were stained with the following. For indirect staining, cyclin B1 (clone GS11, BD Biosciences, San Jose, CA) and cyclin A2 (Beckman Coulter, Brea, CA) antibodies were both used at 0.125 ug in 50 ul volume of PBS-BSA (phosphate buffered saline with 2% bovine serum albumin, Sigma-Aldrich, St. Louis, MO). The secondary antibody, used at 0.25 ug per sample in 50 ul PBS-BSA, was goat anti-mouse conjugated to Alexa Fluor 647 (GAM-A647) from Invitrogen/Molecular Probes (Carlsbad, CA). The cyclin B1 antibody used for direct staining was the same clone described above, conjugated to Alexa Fluor 647 (A647) with kit from Molecular Probes, used according to manufacturer's directions. 0.06 ug in 50 ul PBS-BSA per sample was used for staining. The cyclin A2 antibody used for direct staining was the same clone as above but conjugated to phycoerythrin (Cyclin A2-PE, Beckman Coulter). This was used at 0.125 ug sample. The PHH3 antibody, directly conjugated to Alexa Fluor 488 (#9708, Cell Signaling Technology, Danvers, MA) was used at 0.0125 ug per sample in all reactions. 4′,6-diamidino-2-phenylindole (DAPI) was purchased from Sigma-Aldrich and used at 0.25 ug/ml in PBS (2×10^6^ cells, 500 ul PBS).

### Cytometry

Samples were measured on an LSR II with uv, violet, blue, and red excitation, using stock filters for blue (DAPI), green (A488-PHH3), orange (PE-cyclin A2), and red (A647-cyclin A2 or B1) fluorescence.

### Software

WinList 7.0 (Verity Software House, Topsham, ME) was used for all data file processing and primary analysis. Prism 5.04 (Graphpad, graphpad.com) was used for non-linear regression and plotting expression profiles. Excel 2007 (Microsoft, Redman, WA) was used to generate the multi-gaussian model of a DNA histogram. Prism was used to plot the results. For segmentation of bivariate plots, we used the normal visual/mouse click interface of WinList to set preliminary partial regions that followed the local slope across the bivariate data peak values from each region. We then calculated the vertice positions for bounding lines that would be orthogonal to the local slope. The vertice information was obtained from a hypertext file written by the program. This was manually adjusted to the new calculated vertices. In this manner, we could ensure that the regions were not over or under-lapping. This logic was used for regions where data runs were not orthogonal to an axis. For other region logic, consult the text or Figure legends.

## Supporting Information

Figure S1 **Data preprocessing.** There are several steps to prepare cytometry list mode data for profile extraction. A: removal of aggregates and debris by a 99% contour gate on the singlet population from a plot of pulse width (UV 440-W) vs. integrated pulse (DNA) for the DAPI signal. Arrow points to mitotic events (red dots) that are not included in this gate. These represent some anaphase, telophase, and cytokinetic cells. B: mitotic gate (R3) based on PHH3 fluorescence of cells gated from the Singlet Gate (R2). Background subtraction for cyclin A2 or B1 is accomplished by plotting cyclin fluorescence versus side scatter (SSC-A). The G1 cells are negative for cyclin A2 and partially negative for cyclin B1. We use the compensation mechanism to subtract from all data an amount of cyclin staining related fluorescence that rises in G1 as a function of cell size. C: cyclin A2 data prior to subtraction. D: cyclin A2 data after subtraction. If everything goes correctly, the median fluorescence of G1 cells should be close to zero. To remove some outliers, a restricting gate is applied to a cyclin fluorescence vs. DNA plot of interphase cells (AND gated on R2, NOT gated on R3). This removes doublets, aberrant S phase cells without cyclins, and outliers. E: gate (R5) is set at >99% contour level. F: a final outlier gate is set on interphase cells plus mitotic cells that have been gated ((R2 AND R5) OR R3). The final gating strategy uses ((R2 AND R4 AND R5) OR R3)) to obtain an entire cell cycle distribution of the 2C stem line. Sometimes additional gates are applied on SSC vs. cyclin fluorescence or DNA vs. acquisition time (position in the list). The objective of either of these is to further clean up outliers.
(TIF)

Figure S2 **Two-phase, differential rates of accumulation of both cyclin A2 and B1 expression in interphase can be fit to a complex equations.** Cyclin A2 was fit to a compound equation: *y* = c + b*x* + a*x* ^2^ from 0.38 to 0.7, and c*e^k*x*^ from 0.7 to 0.95. Cyclin B1 was fit to *y* = c1*e ^k1*x*^ from 0.32 to 0.79 and *y* = c2*e ^k2*x*^ from 0.79 to 0.95. Therefore, both cyclins appear to have an early, slow rate and a late, fast rate with cyclin B1 having a more exponential character. The fits were done to specific fluorescence values from single cyclin analyses using experimental procedures that make the amplitude relationships relevant. Therefore, cyclin B1 accumulates at slower rate during S phase than cyclin A2 but at a faster rate in late S/G2. It is also obvious from these plots that cyclin B1 begins accumulation earlier than cyclin A2.
(TIF)
