# Supplementary figures and images for: Dynamic Epitope Expression from Static Cytometry Data: Principles and Reproducibility
Source: PLoS One. 2012 Feb 8;7(2):e30870. doi: 10.1371/journal.pone.0030870 (PMC3275612; doi:10.1371/journal.pone.0030870)

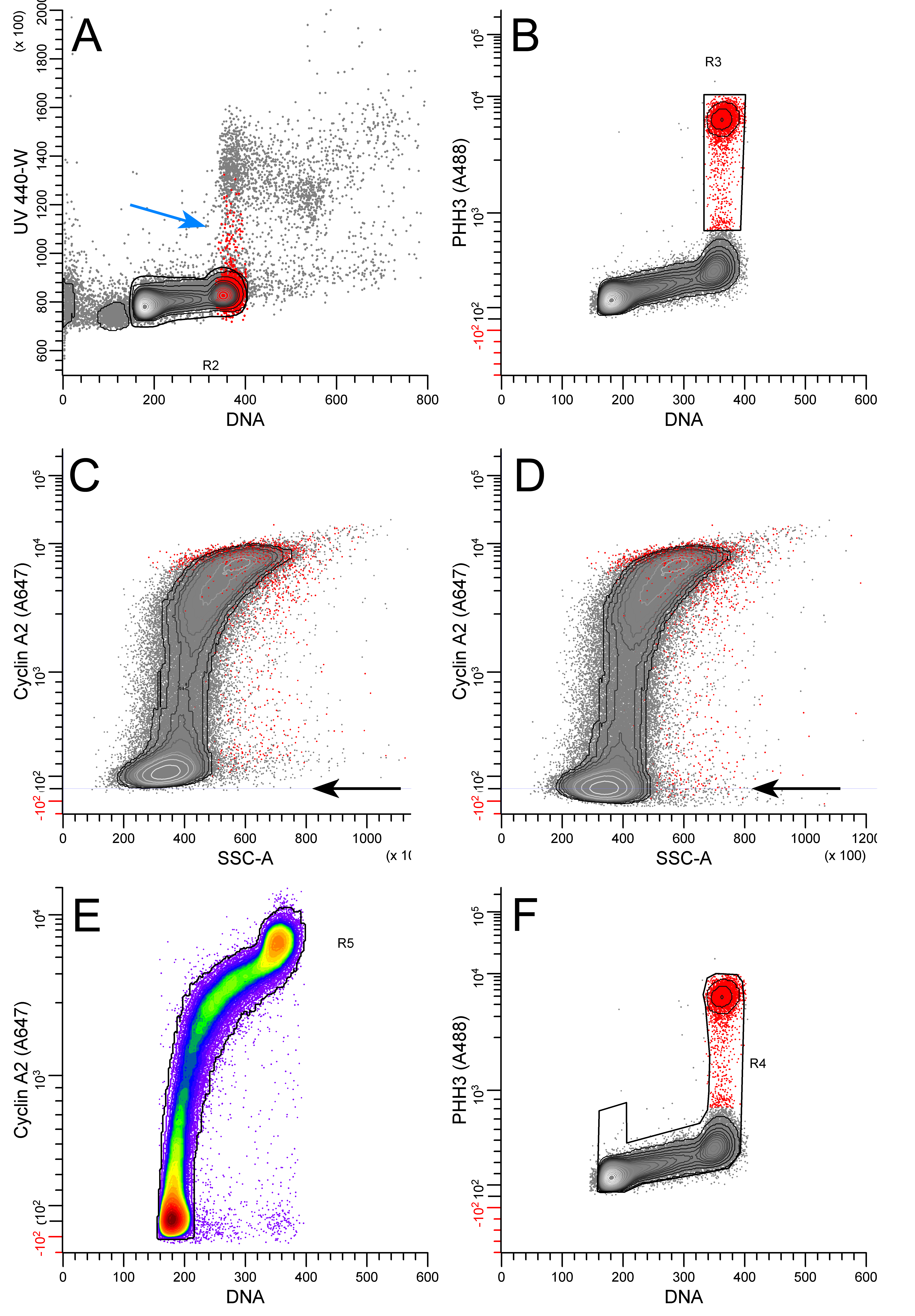

Supplement: Figure S1 — Data preprocessing. There are several steps to prepare cytometry list mode data for profile extraction. A: removal of aggregates and debris by a 99% contour gate on the singlet population from a plot of pulse width (UV 440-W) vs. integrated pulse (DNA) for the DAPI signal. Arrow points to mitotic events (red dots) that are not included in this gate. These represent some anaphase, telophase, and cytokinetic cells. B: mitotic gate (R3) based on PHH3 fluorescence of cells gated from the Singlet Gate (R2). Background subtraction for cyclin A2 or B1 is accomplished by plotting cyclin fluorescence versus side scatter (SSC-A). The G1 cells are negative for cyclin A2 and partially negative for cyclin B1. We use the compensation mechanism to subtract from all data an amount of cyclin staining related fluorescence that rises in G1 as a function of cell size. C: cyclin A2 data prior to subtraction. D: cyclin A2 data after subtraction. If everything goes correctly, the median fluorescence of G1 cells should be close to zero. To remove some outliers, a restricting gate is applied to a cyclin fluorescence vs. DNA plot of interphase cells (AND gated on R2, NOT gated on R3). This removes doublets, aberrant S phase cells without cyclins, and outliers. E: gate (R5) is set at >99% contour level. F: a final outlier gate is set on interphase cells plus mitotic cells that have been gated ((R2 AND R5) OR R3). The final gating strategy uses ((R2 AND R4 AND R5) OR R3)) to obtain an entire cell cycle distribution of the 2C stem line. Sometimes additional gates are applied on SSC vs. cyclin fluorescence or DNA vs. acquisition time (position in the list). The objective of either of these is to further clean up outliers. (TIF) [file pone.0030870.s001.tif]

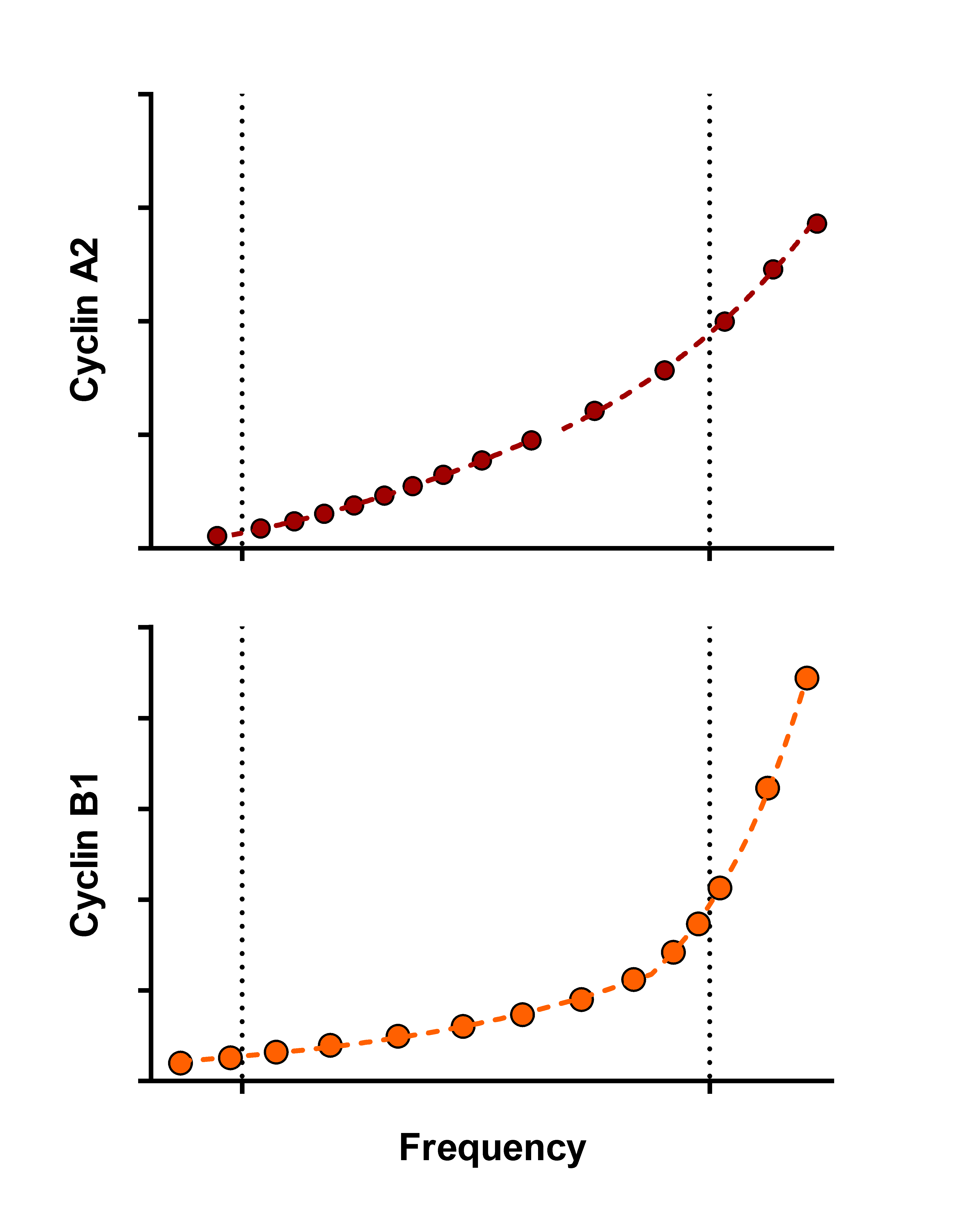

Supplement: Figure S2 — Two-phase, differential rates of accumulation of both cyclin A2 and B1 expression in interphase can be fit to a complex equations. Cyclin A2 was fit to a compound equation: y = c + bx + ax 2 from 0.38 to 0.7, and c*ekx from 0.7 to 0.95. Cyclin B1 was fit to y = c1*e k1x from 0.32 to 0.79 and y = c2*e k2x from 0.79 to 0.95. Therefore, both cyclins appear to have an early, slow rate and a late, fast rate with cyclin B1 having a more exponential character. The fits were done to specific fluorescence values from single cyclin analyses using experimental procedures that make the amplitude relationships relevant. Therefore, cyclin B1 accumulates at slower rate during S phase than cyclin A2 but at a faster rate in late S/G2. It is also obvious from these plots that cyclin B1 begins accumulation earlier than cyclin A2. (TIF) [file pone.0030870.s002.tif]
